# Glycemic control and use of glucose-lowering medications in hospital-admitted type 2 diabetes patients over 80 years

**DOI:** 10.1038/s41598-020-60818-5

**Published:** 2020-03-05

**Authors:** Ditte Resendal Gotfredsen, Siri Vinther, Tonny Studsgaard Petersen, Rikke Cortes, Thomas Bo Jensen, Espen Jimenez-Solem, Mikkel Bring Christensen

**Affiliations:** 1 Department of Clinical Pharmacology, Bispebjerg and Frederiksberg Hospital, Copenhagen, Denmark; 2 Department of Clinical Medicine, University of Copenhagen, Copenhagen, Denmark; 3 Center for Clinical Metabolic Research, Gentofte Hospital, University of Copenhagen, Hellerup, Denmark

**Keywords:** Type 2 diabetes, Epidemiology

## Abstract

Treatment guidelines for type 2 diabetes (T2D) recommend avoidance of hypoglycemia and less stringent glycemic control in older patients. We examined the relation of glycemic control to glucose-lowering medications use in a cohort of patients aged>80 years with a diagnosis of T2D and a hospital admission in the Capital Region of Denmark in 2012–2016. We extracted data on medication use, diagnoses, and biochemistry from the hospitals’ records. We identified 5,172 T2D patients with high degree of co-morbidity and where 17% had an HbA_1c_ in the range recommended for frail, comorbid, older patients with type 2 diabetes (58–75 mmol/mol (7.5–9%)). Half of the patients (n = 2,575) had an HbA_1c_ <48 mmol/mol (<6.5%), and a majority of these (36% of all patients) did not meet the diagnostic criteria for T2D. Of patients treated with one or more glucose-lowering medications (n = 1,758), 20% had HbA_1c_-values <42 mmol/mol (<6%), and 1% had critically low Hba_1c_ values <30 mmol/mol (<4.9%), In conclusion, among these hospitalized T2D patients, few had an HbA_1c_ within the generally recommended glycemic targets. One third of patients did not meet the diagnostic criteria for T2D, and of the patients who were treated with glucose-lowering medications, one-fifth had HbA_1c_-values suggesting overtreatment.

## Introduction

For patients with type 2 diabetes, it is important to maintain blood glucose levels as close to normal as possible in order to reduce the risk of micro- and macrovascular complications^1–4^. Treatment should, however, be individualized according to comorbidities, disease duration, risk of adverse events and in particular hypoglycemia, life expectancy as well as the patient’s own preferences, resources and support system^1^. Elderly people with type 2 diabetes will generally have co-existing illness and relatively few resources^5^. Life expectancy will often be shorter than the time it takes for micro- and macrovascular disease complications to develop and manifest^6,7^. This is in contrast to the potential adverse effects of glucose-lowering medications that often appear in the short term. Hypoglycemia is the most important example of an acute and potentially fatal adverse effect to which elderly are particularly vulnerable^8–15^. Less effective counterregulatory mechanisms, decreased drug elimination, motor and cognitive impairment as well as unspecific/uncharacteristic symptoms all contribute to the heightened risk in elderly patients^16^. Thus, the overall goal with treatment individualization should be to weigh the typically long-term benefits vs. therapy burden and risk of adverse events on the shorter term^7,15,17,18^. Available evidence from the few clinical trials enrolling elderly patients with type 2 diabetes support that the benefits of intensive glycemic control targeting near-normal glycemia may not outweigh potential risks in this population^8,19–22^. This is also reflected in several international guidelines which generally advocate a less stringent treatment approach for older people with coexisting illnesses. An HbA_1c_ target of 58–75 mmol/mol (7.5–9%) after pharmacological intervention, is generally recommended^1,6,7,17^. Recent studies have, however, questioned the extent to which these recommendations have been adopted and implemented in clinical practice^12,23,24^.

Previous studies examining trends in use, effects (glycemic control as measured by HbA_1c_) and harms (e.g. hypoglycemia) of glucose-lowering medications have predominantly focused on the general type 2 diabetes population^25–31^. This study focuses on a cohort of patients aged 80 years or older with a diagnosis of type 2 diabetes and a hospital-based health record in the period 2012–2016. The main objective was to examine glycemic control in relation to use of glucose-lowering medications; secondary objectives included characterizing the patient cohort with regards to comorbidity, drug administration and biochemical status at the time of hospital admission.

## Results

### Patient characteristics and admission diagnoses

A total of 5,172 patients with type 2 diabetes were included in the study (Table 1). The median age was 84 years (IQR 82–88 years) and 54% of the patients were female. Based on Body Mass Index (BMI), 41% were normal weight (BMI 18.5–25 kg/m^2^) and 55% were overweight or obese (BMI >25 kg/m^2^) (Table 1). Regarding biochemical status, LDL-cholesterol was >2,5 mmol/L for 25% of the patients. The estimated glomerular filtration rate (eGFR) was ≤60 mmol/L for 57% of the patients and 56% had a hemoglobin below the reference level calculated for men and women respectively (Table 1). The median duration of hospital admission was four days with pneumonia being the most common cause of admission (4%, n = 211). Diabetes related diagnoses were registered as the primary cause of admission for 2% (n = 78) of all patients and 1% (n = 70) had hypoglycemia as the primary cause of admission.

**Table 1:** Patient characteristics for all patients with type 2 diabetes ≥80 years included in the study.

	n (%)
Unique patients, number	5172 (100%)
**Gender**	
Male	2392 (46%)
Female	2780 (54%)
**Age in years (median, IQR)**	84 (81.5–87.6)
**Days of admission (median, IQR)**	4 (1–9)
**BMI (n = 4139)**	
<18.5	163 (4%)
18.5 – <25	1685 (41%)
25 – <30	1454 (35%)
30 – <40	766 (19%)
≥40	71 (2%)
**Charlson Comorbidity Index**	
0	0 (0%)
1	342 (7%)
2	602 (12%)
>2	4228 (82%)
**HbA**_**1c**_ **(mmol/mol)**	
<30	57 (1%)
30–41	1304 (25%)
42–47	1214 (23%)
48–52	757 (15%)
53–57	544 (11%)
58–74	891 (17%)
≥75	405 (8%)
**LDL (mmol/L)**	**(n = 2983)**
<1.8	1379 (46%)
1.8–2.5	856 (29%)
>2.5	748 (25%)
**Total cholesterol (mmol/L)**	**(n = 2222)**
<5	1820 (82%)
≥ 5	402 (18%)
**HDL (mmol/L)**	**(n = 3105)**
≤1	842 (27%)
>1	2263 (73%)
**Creatinine (normal range men: 50–90, women: 60–105) (µmol/L)**	**(n = 5154)**
Within range	2386 (46%)
Above range	2541 (49%)
Under range	227 (4%)
**eGFR (mL/min/1,73m**^**2**^**)**	**(n = 4221)**
≤60	2426 (57%)
>60	1795 (43%)
**Haemoglobin (normal range women: 7.3–9.5, men: 8.3–10.5) (mmol/L)**	**(n = 5118)**
Within range	2191 (43%)
Above range	64 (1%)
Under range	2863 (56%)
**TSH (normal range 0.35–4.0 or 0.65–4.80) (IU/L)**	**(n = 3862)**
Within range	3278 (85%)
Above range	295 (8%)
Under range	289 (7%)

Values are displayed in absolute numbers, percentages and median (inter-quartile range). For haemoglobin and creatinine, the reference values are displayed for men and women separately.

### Comorbidities

The majority (82%, n = 4,228) of patients had a high level of comorbidity with a value >2 on the Charlson Comorbidity Index (Table 1). Detailed data on the cognitive status of the patients was not available, but 16% had a diagnosis of dementia (Table 2). Hypertension was the most commonly registered comorbidity (71%), followed by congestive heart failure (32%), peripheral vascular disease (18%) and previous myocardial infarction (13%) (Table 2).

**Table 2:** Number of patients with co-morbidities, using all available data for each individual.

Co-morbidities	n (%)
Hypertension	3648 (71%)
Atrial fibrillation	1990 (38%)
Congestive heart failure	1650 (32%)
Cerebrovascular disease	1547 (30%)
Chronic pulmonary disease	1207 (23%)
Moderate to severe renal disease	1079 (21%)
Non-skin malignancy	984 (19%)
Peripheral vascular disease	930 (18%)
Dementia	831 (16%)
Myocardial infarction	689 (13%)
Thyroid disorders	524 (10%)
Depression	455 (9%)
Peptic ulcer disease	400 (8%)
Rheumatologic disease	137 (3%)
Metastatic solid tumor	124 (2%)
Moderate or severe liver disease	41 (1%)
Schizophrenia	10 (0%)

### Glycemic control

**Figure 1:**
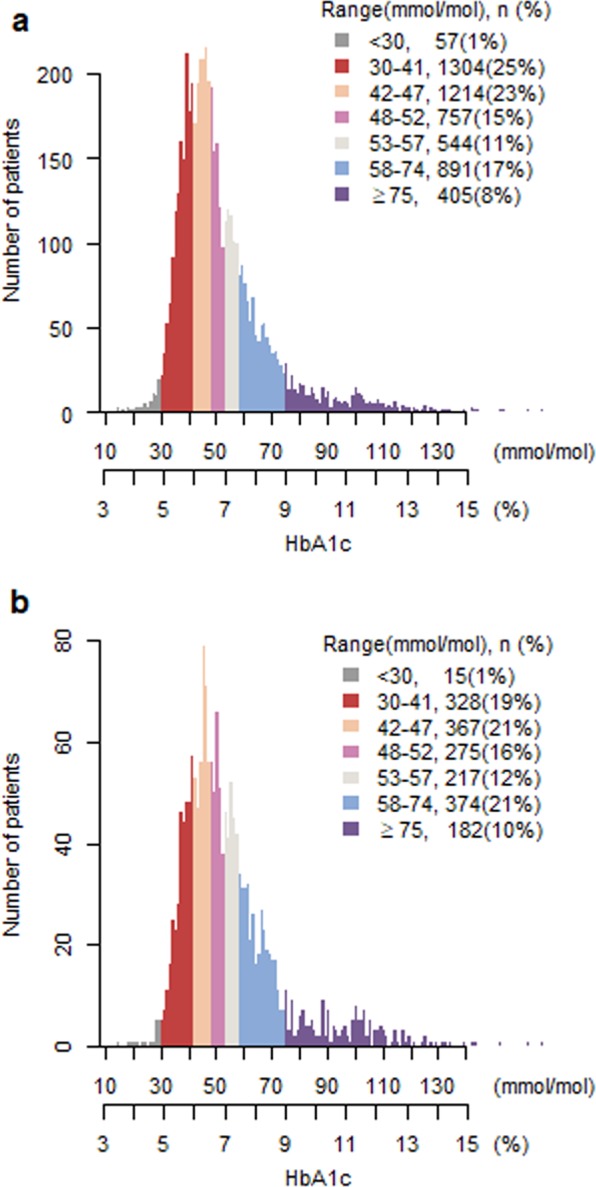
Distribution of individual HbA_1c_-values for patients with type 2 diabetes >80 years with and without glucose-lowering medications divided into HbA_1c_-categories. HbA_1c_-values on the x-axis are displayed in both percentage and in mmol/mol. (**a**) Displays HbA_1c_-values for all patients (n = 5172). (**b**) Displays HbA_1c_-values for patients treated with glucose-lowering medications (n = 1758).

The distribution of HbA1c values is shown in Fig. 1. Most patients (91%, n = 4,710) had an HbA_1c_ between 30–75 mmol/mol (4.9–9%). Half of the patients (n = 2,575) had an HbA_1c_ <48 mmol/mol (<6.5%), and 26% (n = 1,361) had an HbA_1c_ <42 mmol/mol (<6%). In the other end of the spectrum, 8% (n = 405) had HbA_1c_-values >75 mmol/mol (>9%) (Table 1). A total of 17% (n = 891) had an HbA_1c_ between 58–75 mmol/mol (7.5–9%), i.e. within the interval recommended for elderly, comorbid patients with overt type 2 diabetes (Table 1).

### Glucose-lowering medications

Close to one third of patients (34%, n = 1,758) were treated with at least one glucose-lowering medication at discharge (Table 3); 41% (n = 2,100) were administered at least one glucose-lowering medication, including sliding scale bolus insulin, during the index hospital admission (data not shown). Among the patients treated with glucose-lowering medication at discharge, one fourth (25%, n = 448) were treated with two or more glucose-lowering medications (Table 3). The most commonly used glucose-lowering medications were metformin (50%), basal insulin (32%), bolus insulin (10%), sulphonylureas (14%) and dipeptidyl peptidase-4 inhibitors (14%) (Table 4).

**Table 3:** Number of patients grouped by number of glucose-lowering medications administered at the time of hospital discharge and HbA_1c_-value (obtained ±90 days before hospital admission).

Number of glucose-lowering medications	HbA_1c_							
	<30	30–41	42–47	48–52	53–57	58–74	75+	Total
0	42 (74%)	976 (75%)	847 (70%)	482 (64%)	326 (60%)	518 (58%)	223 (55%)	3414 (66%)
1	12 (21%)	279 (21%)	284 (23%)	217 (29%)	155 (29%)	250 (28%)	113 (28%)	1310 (25%)
2	2 (4%)	43 (3%)	75 (6%)	50 (7%)	55 (10%)	103 (12%)	60 (15%)	388 (8%)
3	0 (0%)	5 (0%)	8 (1%)	7 (1%)	7 (1%)	19 (2%)	8 (2%)	54 (1%)
4	1 (2%)	1 (0%)	0 (0%)	1 (0%)	0 (0%)	2 (0%)	1 (0%)	6 (0%)
Total	57 (100%)	1304 (100%)	1214 (100%)	757 (100%)	543 (100%)	892 (100%)	405 (100%)	5172 (100%)

HbA_1c_-values are divided into categories and displayed in mmol/mol.

**Table 4:** Antidiabetic medication at the time of hospital discharge in relation to HbA_1c_-values (obtained ±90 days before hospital admission) for very old patients with type 2 diabetes.

Type of glucose-lowering medication	HbA_1c_							
	<30	30–41	42–47	48–52	53–57	58–74	75+	Total
Acarbose	0 (0%)	0 (0%)	0 (0%)	0 (0%)	2 (100%)	0 (0%)	0 (0%)	2 (100%)
Basal Insulin	1 (0%)	51 (9%)	64 (11%)	75 (13%)	79 (14%)	188 (33%)	105 (19%)	563 (100%)
Bolus Insulin	3 (2%)	26 (15%)	23 (14%)	21 (12%)	28 (17%)	44 (26%)	24 (14%)	169 (100%)
DPP-4i	2 (1%)	30 (12%)	56 (22%)	34 (13%)	36 (14%)	56 (22%)	38 (15%)	252 (100%)
GLP-1 RA	0 (0%)	5 (16%)	7 (23%)	7 (23%)	1 (3%)	10 (32%)	1 (3%)	31 (100%)
Metformin	10 (1%)	204 (21%)	254 (26%)	163 (17%)	110 (11%)	169 (17%)	75 (8%)	985 (100%)
SGLT-2i	0 (0%)	2 (12%)	1 (6%)	2 (12%)	4 (24%)	6 (35%)	2 (12%)	17 (100%)
SU	4 (2%)	66 (26%)	53 (21%)	40 (16%)	26 (10%)	48 (19%)	16 (6%)	253 (100%)
Total	20 (1%)	384 (17%)	458 (20%)	342 (15%)	286 (13%)	521 (23%)	261 (11%)	2272 (100%)

Values are displayed in absolute numbers. HbA_1c_-values are divided into categories and displayed in mmol/mol. Patients count more than once if administered more than one kind of antidiabetic.

DPP-4i: dipeptidylpeptidase-4 inhibitor, SGLT-2i: sodium-glucose cotransporter-2 inhibitor, SU: sulfonylurea, GLP-1RA: Glucagon-like peptide-1 receptor agonist.

### Glucose-lowering medications in relation to glycemic control

Of those treated with a glucose-lowering medication at discharge (n = 1,758), close to half 48%, n = 844) had an HbA_1c_ within the interval recommended for elderly without significant comorbidity (43–57 mmol/mol (6.0–7.5%)). One third had higher HbA1c-values, 21% (n = 374) had a Hba1c between 58–74 mmol/mol (7.5–9), and 10% (n = 182) had Hba1c >75 mmol/mol (9%); while the remaining 20% (n = 343) had near-normalized Hba1c (<42 mmol/mol (6%)) while continuing glucose-lowering medication at discharge. Of the patients with near-normalization of Hba1c values, 15% (n = 52) took two or more glucose-lowering medications (Table 3, Fig. 1) most frequently metformin, insulin and sulphonylureas (Table 4). One percent (n = 15) of the patients treated with an glucose-lowering medication at discharge had very low Hba_1c_-values <30 mmol/mol (<4.9%) (Table 3, Fig. 1).

For those patients who did not receive a glucose-lowering medication at discharge (n = 3,414), 55% (n = 1,865) had HbA_1c_-values that did not justify a diagnosis of type 2 diabetes (i.e. HbA_1c_ <48 mmol/mol (<6,5%)) (Table 3, Fig. 1). At the other end of the spectrum, 7% (n = 223) had Hba1c levels for which glucose-lowering medications are generally recommended (i.e.>75 mmol/mol (9%)).

## Discussion

Based on hospital electronic health records covering the entire population of the Capital Region of Denmark (1.8 million inhabitants) from 2012 to 2016, we investigated the demographics and the degree of glycemic control in relation to glucose-lowering medications in patients with type 2 diabetes aged 80 years or more. Our main findings were (1) almost half of the patients had an HbA_1c_<48 mmol/mol (<6,5%), and of these 72% (n = 1865, 36% of all patients) were not treated with a glucose-lowering medication and thus did not fulfil the diagnostic criteria of type 2 diabetes; (2) of the patients treated with one or more glucose-lowering medications (often including insulin and/or sulphonylureas), 20% had HbA_1c_-values below 42 mmol/mol (6%) and 1% had critically low HbA_1c_ values <30 mmol/mol (<4.9%), indicating overtreatment. Conversely, 8% of all patients had Hba1c values >75 mmol/mol (>9%), indicating possible undertreatment.

A surprising finding was that based on HbA_1c_-value, 36% (n = 1,865) of all the admitted patients did meet the criteria for their diagnosis of type 2 diabetes. The diagnoses were all registered by a physician authorized in Denmark and could have been registered many years prior to the index admission. Thus, one potential explanation for our finding could be that type 2 diabetes is not a chronic disease but rather a condition that may in some cases remit with old age – a notion that has been proposed before^32,33^. Hence, Abdelhafiz *et al*. proposed that frailty among older people with type 2 diabetes might lead to the remission of type 2 diabetes with the suggested mechanisms being weight loss accompanied by reduced amounts of visceral fat and thereby improved insulin sensitivity^32^. Such a mechanism bears resemblance to that described for patients having bariatric surgery and/or substantial weight loss and afterwards experience remission of their type 2 diabetes^34,35^.

We report that only 17% of included patients had an HbA_1c_ between 58–75 mmol/mol (7.5–9%), the interval generally recommended for elderly with significant comorbidities and limited life expectancy. That our patients were indeed highly comorbid is evidenced by the Charlson comorbidity score, where 94% scored 2 or more^36^. Of those with an HbA_1c_ <42 mmol/mol (<6.0%), 25% were treated with one or more glucose-lowering medications. These findings are in line with findings from other studies that have raised concerns about the potential overtreatment of older people with type 2 diabetes^12,23,24,37–39^. Among these is a large register-based study by Tseng *et al*. including 652,738 patients from the Veteran Health Administration. They reported that approximately 50% of patients aged 75 years or older, who were treated with insulin and/or sulphonylureas, had an HbA_1c_ <53 mmol/mol (<7%)^12^. Similarly, results from The Fremantle Diabetes Cohort Study, which included 367 patients over the age of 75 with type 2 diabetes showed that approximately three of five (61%) of the patients had an HbA_1c_ <53 mmol/mol (<7%)^37^. As treatment needs to be individualized according to a patient’s preferences and resources as well as life expectancy it is of interest that in our cohort dementia was registered as a diagnosis for 16% and non-skin malignancy for 19% of the included patients. Studies of frail patients with type 2 diabetes and limited life expectancy, such as nursing home residents, have suggested that particularly elderly with dementia are overtreated with glucose-lowering medications. Thus, in a nursing home population, 46–74% of the patients had an HbA_1c_ <53 mmol/mol (<7%)^24,39,40^. Although the distributions of Hba_1c_-values in the mentioned nursing home studies were similar to ours, cognitive and functional impairment may be more frequent in the nursing home setting. One percent (n = 70) of our population had hypoglycemia as the primary cause of admission. However, this is likely an underestimate of the number of patients at high risk of hypoglycemia. In older people, hypoglycemia can go undiscovered and be difficult to recognize due to unspecific symptoms^11^. Thus, the substantial proportion of patients, who in the context of near-normal Hba1c (i.e. below 42 mmol/mol (6%)) continued treatment with a sulphonylurea (n = 70) or insulin (n = 82) could be considered at high risk of hypoglycemic events^11,16^. Thus, our study adds to the evidence suggesting that the recommendations favoring looser glycemic control in elderly, comorbid people similar to our population has not been fully adopted into clinical practice.

Our study has important strengths such as the large sample size, the high data quality from rather accurate national registers with the possibility of linking biochemical data with health record data and drug use. Nonetheless, this register-based study also has some limitations. In our study, only 34% of elderly patients with a diagnosis of type 2 diabetes were treated with glucose-lowering medications. Other studies on glycemic control in older people, including the mentioned studies of nursing home residents and larger cohort studies report a much higher proportion of patients treated with glucose-lowering medication. Thus, between 85–100% of the patients received glucose-lowering medication in other cohort studies of a general population with type 2 diabetes^12,23,37^, and up to 86% were pharmacologically treated in studies investigating glycemic control in nursing home residents^24,39,40^. Our lower treatment prevalence is most likely due to the fact that many patients in our cohort did not meet the criteria for type 2 diabetes at the time of study. Since our study was based on a cohort identified by a hospital admission, and data analyses were limited to the time around hospital admission, we did not have information on the duration of diabetes or the glycemic control and use of antidiabetic medication over time. Access to this information could have strengthened our interpretation particularly the reason for the high proportion of patients not fulfilling the diagnostic criteria for type 2 diabetes. There is some indication that our cohort does not fully reflect the population in the capital region of Denmark. Thus, in our cohort, 54% were female, while the concurrent female proportion in the general population was 65%. The reason for such relative underrepresentation of females in our cohort is unclear. Another issue is that 56% had a hemoglobin below reference level, which theoretically could lead to an underestimation of the HbA_1c_-values. However, as proposed by samples from another Danish population, mild to moderate anemia does not seem have significant impact on the interpretation of HbA_1c_-values^41^.

In this hospital-based cohort consisting of more than 5000 patients, few patients ≥80 years with type 2 diabetes had an HbA_1c_ within the limits generally recommended for this population. Many patients were not treated with glucose-lowering medications and had HbA_1c_-values that could not justify a diagnosis of type 2 diabetes. Of those treated with one or more glucose-lowering medications, quite many had either high or low HbA_1c_-values, suggesting under- and overtreatment, respectively. Our study supports the assumption that a diagnosis of type 2 diabetes may remit with age. Moreover, it suggests that recommendations for glycemic control in elderly patients with type 2 diabetes are not fully implemented in clinical practice.

## Methods

### Study cohort and data sources

The study was a retrospective cohort study using data from the Capital Region of Denmark from January 1, 2012 to May 15, 2016. We analyzed the first hospital admission for each patient, where an HbA_1c_ measurement in proximity to the hospital admission (±90 days) was available. On admission, patients were required to be at least 80 years of age and have a prior diagnosis of type 2 diabetes (ICD-10 code DE11). Diagnoses were obtained from the regional system feeding data to The Danish National Patient Register^42^. Drug utilization was obtained from The Electronic Patient Medication module, which is a database for in-hospital drug-use in the Capital Region of Denmark^43^. HbA_1c_-values, as well as biochemical status (blood lipids (cholesterol, LDL and HDL), kidney function (creatinine, eGFR), hemoglobin levels and TSH), were gathered from The Clinical Laboratory Information System^44^. Body Mass Index (BMI) was obtained from the medical health records. Data sources were linked using the unique and permanent Danish identification number^45^.

### Exposure and comorbidity

Exposure to a glucose-lowering medication was defined as an active prescription of a glucose-lowering medication (Anatomical Therapeutic Chemical classification (ATC)-code A10) at the time of discharge from the hospital and with at least one administration during the hospital admission. To evaluate patient comorbidity, we used diagnoses to calculate The Charlson Comorbidity Index, which is a measure of comorbidity burden and has been shown to be correlated with life expectancy^36^.

### Statistical methods

Data are presented using standard descriptive statistics including median and interquartile ranges. Data management was conducted using R^46^.

### Ethics

According to the Danish “Act on Research Ethics Review of Health Research Projects” section 14 (2), retrospective register-based studies do not require ethical approval in Denmark. The study was approved by The Danish Data Protection Agency (BFH-2016–058, I-Suite nr.: 04906) and The Danish Patient Safety Authority (3-3013-1884/1/).

### Compliance with ethics guidelines

This article is based on previously conducted health data and does not contain any studies with human participants or animals performed by any of the authors.

## Data Availability

The dataset used in this study is not available due to local law.
